# Perinatal mental health outcome measures in a mother and baby unit

**DOI:** 10.1192/bjo.2021.290

**Published:** 2021-06-18

**Authors:** Johanna O'Connor, Raunica Katyal

**Affiliations:** 1 Norfolk & Norwich University Hospital; 2 Z Green- Thompson, Norfolk and Suffolk NHS Foundation Trust

## Abstract

**Aims:**

To Audit Perinatal outcome measures and understand better the population served in order to improve care and understand risks. Our audit standards inculded: paired HoNOS and PBQ recorded on admission and discharge as well as ASQ scores prior to admission.

**Method:**

Health of the Nation Outcome Scales (HoNOS), Postpartum Bonding Questionnaire (PBQ) and Ages and Stages Questionnaires (ASQ) were recorded on Lorenzo and SystmOne. Scores were collected over 20 months within the same MBU and these were analyzed.

**Result:**

Our audit standards had an overall audit compliancy of 73% with paired HoNOS better than PBQ. Mental health severity mitigated and maternal bonding improved to a significant degree. Depression was the principal presentation as were patients from deprived areas. Only 55% of babies had ASQ scores completed appropriately pre- admission.

**Conclusion:**

As the newest MBU in the country, this an initial foray of perinatal outcomes. Gratifyingly, benefits of MBU admission for mother and baby is evidenced in this snapshot.

